# One-Year Results of Photodynamic Therapy Combined with Intravitreal Ranibizumab for Exudative Age-Related Macular Degeneration

**DOI:** 10.1155/2012/154659

**Published:** 2011-12-07

**Authors:** Tomoko Nakamura, Akio Miyakoshi, Kazuya Fujita, Tatsuya Yunoki, Keiichi Mitarai, Shuichiro Yanagisawa, Chiharu Fuchizawa, Atsushi Hayashi

**Affiliations:** Department of Ophthalmology, Graduate School of Medicine and Pharmaceutical Sciences, University of Toyama, 2630 Sugitani, Toyama 930-0194, Japan

## Abstract

*Purpose*. To evaluate the effects of photodynamic therapy (PDT) combined with intravitreal injection of ranibizumab (IVR) for exudative age-related macular degeneration (AMD). *Methods*. Retrospective case series. Thirty eight eyes of 38 patients with exudative AMD underwent combined therapy consisting first of IVR, followed by PDT within a week and the second IVR at 1 month. All patients were followed up for more than 12 months. The best corrected visual acuity (BCVA) and central macular thickness (CMT) were examined. *Results*. The mean number of IVR and PDT sessions were 2.9 ± 1.3 and 1.1 ± 0.3, respectively. The mean BCVA and CMT were significantly improved to 0.38 logMAR units (*P* < 0.01) and 240 *μ*m (*P* < 0.01) at 12 months, respectively. Thirty-six of 38 eyes (94.8%) improved or maintained BCVA at 12 months. *Conclusion*. PDT combined with IVR for exudative AMD was effective at improving visual acuity and CMT with a low recurrence rate for 12 months.

## 1. Introduction

Age-related macular degeneration (AMD), especially exudative AMD is a major cause of visual disability and blindness [1]. Photodynamic therapy (PDT) has been shown to occlude the neovascular membrane and decrease visual acuity loss in exudative AMD [1, 2]. However, several studies have shown that PDT causes damage to the physiological choriocapillary layer, and that repeated PDT therapy often leads to occlusion of the choriocapillaris [2, 3].

Recently, monthly intravitreal injection therapy of antivascular endothelial growth factor (VEGF) antibody has been shown to increase visual acuity in exudative AMD [4–7]. Because the pathogenesis of choroidal neovascularization (CNV) is complex and exudative AMD is a multifactorial disease, combined therapy adopting different mechanisms to inhibit and destroy CNV, namely, PDT and anti-VEGF antibody therapy, may facilitate improvement of visual acuity on exudative AMD.

Husain et al. reported that PDT combined with intravitreal injections of ranibizumab showed a greater reduction of fluorescein leakage compared to PDT only in monkey eyes [8]. Several clinical studies have shown that a combination therapy of PDT and ranibizumab effectively reduced the number of intravitreal injections compared to anti-VEGF antibody monotherapy regimens [9–13].

In this study, we examined efficacy of PDT combined with intravitreal injections of ranibizumab (IVR) in patients with exudative AMD and compared results of typical AMD and polypoidal choroidal vasculopathy (PCV).

## 2. Materials and Methods

Thirty-eight eyes of 38 consecutive Japanese patients (29 men, 9 women) who were diagnosed with exudative AMD without previous treatment at Toyama University Hospital between April and December 2009 were enrolled in this study. The study was conducted in accordance with the Institutional Guidelines of the University of Toyama and was approved by the Institutional Review Board. The procedures conformed to the tenets of the World Medical Association's Declaration of Helsinki. 

The inclusion criteria for this study were patient age older than 50 years, exudative AMD in the macular area, decimal best-corrected visual acuity (BCVA) of 0.7 or worse, and a greatest linear dimension (GLD) of 5400-*μ*m or less. The exclusion criteria were CNV caused by other diseases, previously received subfoveal laser treatment, verteporfin photodynamic therapy, or experimental treatments for exdative AMD. Patients who had uncontrolled hypertension, a recent myocardial infarction, or cerebral vascular accidents were also excluded. 

Before the initial treatment, all patients underwent baseline ophthalmic examinations, which included decimal BCVA, fundus examination, optical coherence tomography (OCT) using an RTVue-100 (Optovue Inc., Fremont, Calif, USA), fluorescein angiography (FA), and indocyanine green angiography (ICGA). 

All patients were followed up for more than 12 months. At each visit, a fundus examination, BCVA, and OCT were performed. FA and ICGA were performed when recurrence of leakage was suspected by OCT and clinical findings during the followup examinations.

 Patients received one intravitreal injection of 0.5 mg ranibizumab (IVR), followed by PDT within a week after the first IVR. This treatment schedule was followed by Sato et al. [14]. A standard-fluence verteporfin PDT was performed at an infusion dosage of 6 mg/m^2^. Application of a 689-nanometer laser at a dose of 50 J/cm^2^ was started 15 min after the start of verteporfin infusion using a Visulas PDT system 690S (Carl Zeiss Meditec AG, Jene, Germany). A second IVR was given to all eyes at 4 weeks after the first IVR.

Additional treatments were determined by BCVA, CMT, and FA with ICGA. The retreatment criteria for IVR were as follows; (1) decrease of BCVA by more than 0.1 logMAR unit, (2) more than 100-*μ*m of increase in central macular thickness (CMT), (3) the presence of subretinal fluid or intraretinal edema at the fovea on OCT, (4) new sub- or intraretinal hemorrhage, or (5) signs of active CNV leakage on fluorescein angiography. These retreatment criteria were referred to the TROPEDO trial [12]. If the CNV was increased in size or relapsed, additional PDT was performed. 

We calculated GLD in each eye based on the findings of FA for PDT treatment. In case of polypoidal choroidal vasculopathy (PCV), ICGA was utilized for GLD calculation. The sizes of GLD were divided into 3 groups; GLD ≦1800 *μ*m, GLD between 1801 and 3500 *μ*m, and GLD between 3501 and 5400 *μ*m group. We evaluated BCVA and CMT in GLD groups.

CMT was manually measured at the fovea with OCT images. We also evaluated the junction line between the inner and outer segments of the photoreceptors (IS/OS) beneath the fovea at baseline and 12 months with OCT images. We used a grading scale reported by Mitamura et al. [15]. IS/OS was categorized into 3 grades, which were grade 0 (invisible IS/OS line), grade 1 (abnormal or discontinuous IS/OS line), and grade 2 (normal or well-preserved IS/OS line). Improvement of BCVA was defined as a decrease of more than 0.3 logMAR units, and deterioration of BCVA was defined as an increase of more than 0.3 logMAR units. 

Hypoperfusion of the choriocapillaris caused by PDT was evaluated with ICGA at 3 months after the first PDT according to a grading scale by Michels et al. [16].

Statistical analyses were performed with JMP 9 (SAS Institute, Cary, NC, USA). Data analyses included a paired *t*-test, nonparametric correlation analysis (Spearman), and one-factor analysis of variance (ANOVA). *P* values less than 0.05 were considered statistically significant.

## 3. Results

### 3.1. Baseline Characteristics and Followup

The mean age of the 38 patients (mean ± standard deviation) was 72.4 ± 8.9 years (range: 53–90 years). The mean followup term was 14.0 ± 1.7 months (range: 12–17 months). Based on findings of FA and ICGA at baseline, lesion types of CNV of 38 eyes were divided into 10 eyes (26.3%) of predominantly classic CNV, 6 eyes (15.8%) of minimally classic CNV, 8 eyes (21.1%) of occult CNV, and 14 eyes (36.8%) of polypoidal choroidal vasculopathy (PCV). 

According to the GLD size, 24 eyes of typical AMD were divided into 10 eyes of the group of GLD ≦1800 *μ*m, 11 eyes of the group of GLD between 1801 and 3500 *μ*m, and 3 eyes of the group of GLD between 3501 and 5400 *μ*m. Fourteen eyes of PCV were divided into 5 eyes of the group of GLD ≦1800 *μ*m, 5 eyes of the group of GLD between 1801 and 3500 *μ*m, and 4 eyes of the group of GLD between 3501 and 5400 *μ*m. 

Each patient received an average of 1.1 ± 0.3 (range: 1-2) sessions of PDT and 2.9 ± 1.3 (range: 2–6) IVR during 12-month followup (Table 1). Twenty-two of 38 eyes (57.9%) were sufficiently healed by the initial treatment with a single PDT and two IVR. Sixteen of 38 eyes (42.1%) underwent an additional one- to- four IVR. Five of 38 eyes (13.2%) required another PDT session. Once the CNV lesion achieved scarred by one or two PDT sessions combined with 2- to- 6 IVR, a recurrence of exudative changes at the fovea was detected in 4 eyes (10.5%) during the 12-month followup (Table 2). As a retreatment for these 4 recurrent eyes, 1 eye underwent additional IVR and 3 eyes underwent another PDT session with IVR.

### 3.2. Visual Acuity

Mean BCVA of all 38 eyes was 0.58 ± 0.40 logMAR units at baseline, 0.47 ± 0.40 at 1 month, 0.33 ± 0.28 at 3 months, 0.35 ± 0.38 at 6 months, and 0.38 ± 0.39 at 12 months. Mean BCVA was significantly improved at 1, 3, 6, and 12 months, compared with the baseline (*P* < 0.01, resp.). 

**Figure 1:**
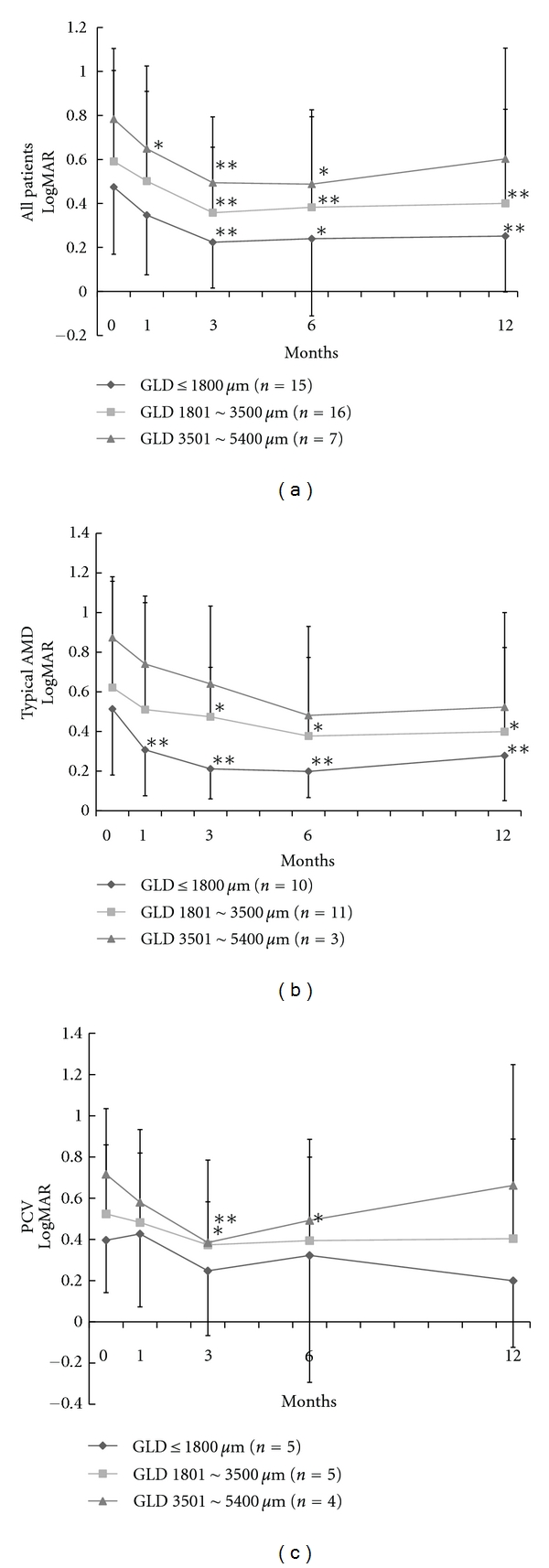
Changes in mean best corrected visual acuity (BCVA). Visual acuity was expressed as the logarithm of minimal angle of resolution (logMAR). (a) Mean BCVA of all 38 patients in three GLD groups; GLD ≦ 1800 *μ*m, GLD between 1801 and 3500 *μ*m, and GLD between 3501 and 5400 *μ*m. The group of greatest linear dimension (GLD) ≦ 1800 *μ*m showed better BCVA than those of the other 2 groups at all time points. However, there was no significant different among the 3 groups (*P* > 0.05). (b) Mean BCVA of typical AMD of 24 patients in three GLD groups. (c) Mean BCVA of PCV of 14 patients in 3 GLD groups. Bars indicate standard deviations. *P < 0.05, **P < 0.01; *P* value for comparison between baseline and each visit. AMD: age-related macular degeneration, PCV: polypoidal choroidal vasculopathy.

Figure 1 shows the changes in visual acuity according to GLD groups. In all 3 groups, the mean BCVA was improved at 3 and 6 months after the combined therapy, compared with the baseline (Figure 1(a)). The group of GLD ≦ 1800 *μ*m showed better BCVA than groups of GLD between 1801 and 3500 *μ*m and GLD between 3501 and 5400 *μ*m at all time points, however, no statistically significant difference was detected among the three groups (*P* > 0.05, Figure 1(a)). As shown in Figure 1(b), the group of GLD ≦ 1800 *μ*m showed better mean BCVA than the other 2 groups in the eyes with typical AMD. Mean BCVA of the group of GLD ≦ 1800-*μ*m was significantly improved at 1, 3, 6, and 12 months (*P* < 0.01), compared with the baseline. However, no statistical difference was detected among the 3 groups. As shown in Figure 1(c), the group of GLD ≦ 1800 *μ*m showed better mean BCVA than the other 2 groups in the eyes with PCV, however, no statistical difference was also detected. 

Table 3 showed changes in BCVA from the baseline in all 38 eyes during 12 months. The number of eyes with improved or stable BCVA at 12 months compared with the baseline was 36 (94.7%). The visual acuity of the remaining 2 eyes (5.3%) had deteriorated more than 0.3 logMAR units at 12 months. Reasons for the decreased visual acuity of these 2 eyes were the movement of subretinal hemorrhage to beneath the fovea in 1 eye of PCV and the recurrence of subfoveal CNV at 11 months in 1 eye.

### 3.3. Central Macular Thickness

The mean CMT of all 38 eyes was 414±120 *μ*m at baseline, 279 ± 126 *μ*m at 1 month, 225 ± 74 *μ*m at 3 months, 247 ± 120 *μ*m at 6 months, and 240 ± 91 *μ*m at 12 months. The mean CMTs at 1, 3, 6, and 12 months were significantly decreased compared with the baseline (*P* < 0.01, resp.). 

**Figure 2:**
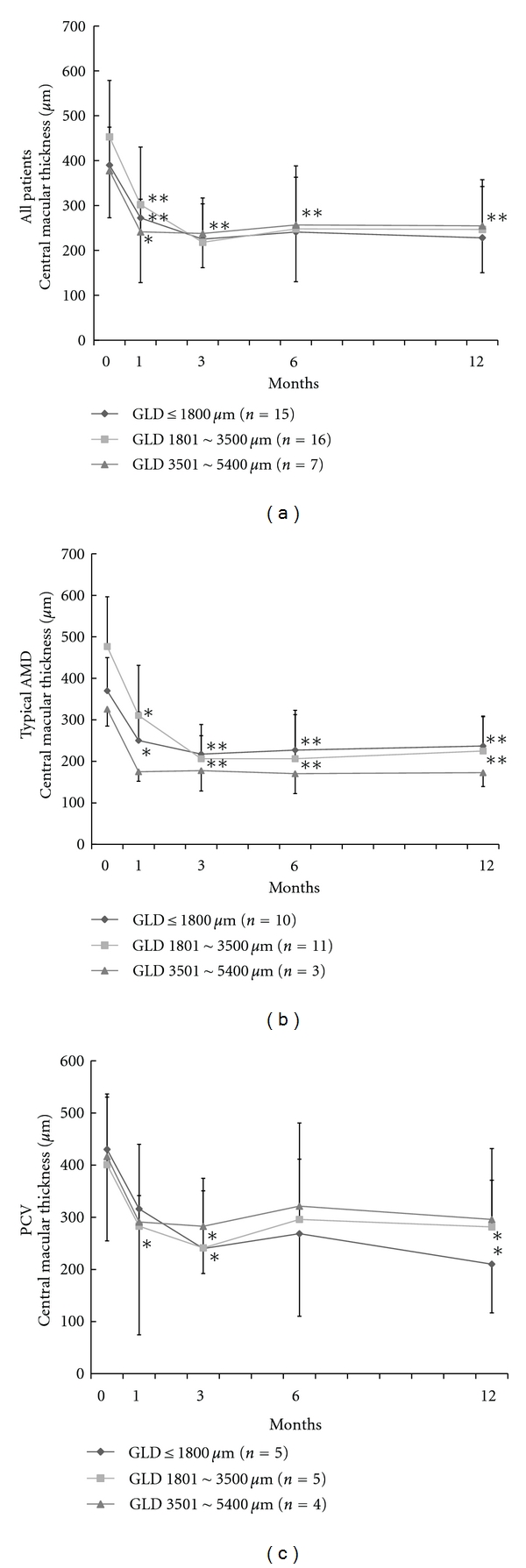
Changes in mean central macular thickness (CMT). (a) Mean CMT of all 38 patients in three GLD groups; GLD ≦ 1800 *μ*m, GLD between 1801 and 3500 *μ*m, and GLD between 3501 and 5400 *μ*m. All 3 groups showed similar values among the 3 groups at all time points. (b) Mean CMT of typical AMD of 24 patients. (c) Mean CMT of PCV of 14 patients in 3 GLD groups. There was no significant difference among the three groups (*P* > 0.05). Bars indicate standard deviations. *P < 0.05, **P < 0.01; *P* value for comparison between baseline and each visit. AMD: age-related macular degeneration, GLD: greatest linear dimension. PCV: polypoidal choroidal vasculopathy.

Changes in the mean CMT of 38 eyes of the 3 GLD groups were shown in Figure 2(a). All groups significantly decreased the mean CMT after the combined therapy (*P* < 0.01) and showed similar mean CMTs among the 3 groups after 3 months. There was no significant difference among the 3 groups. As shown in Figure 2(b), the mean CMT in the eyes with typical AMD significantly decreased after 1 month in the groups of GLD≦ 1800 *μ*m and GLD between 1801 and 3500 *μ*m (*P* < 0.01, *P* < 0.05), compared with the baseline. However, the mean CMT in the group of GLD between 3501 and 5400 *μ*m did not significantly decrease compared with the baseline. As shown in Figure 2(c), the mean CMT in the eyes with PCV was significantly decreased at 1, 3, and 12 months (*P* < 0.05) in the groups of GLD≦ 1800 *μ*m and GLD between 1801 and 3500 *μ*m, compared with the baseline. However, there was no significant difference in the changes of mean CMT in the group of GLD between 3501 and 5400 *μ*m. 

### 3.4. IS/OS Line

**Figure 3:**
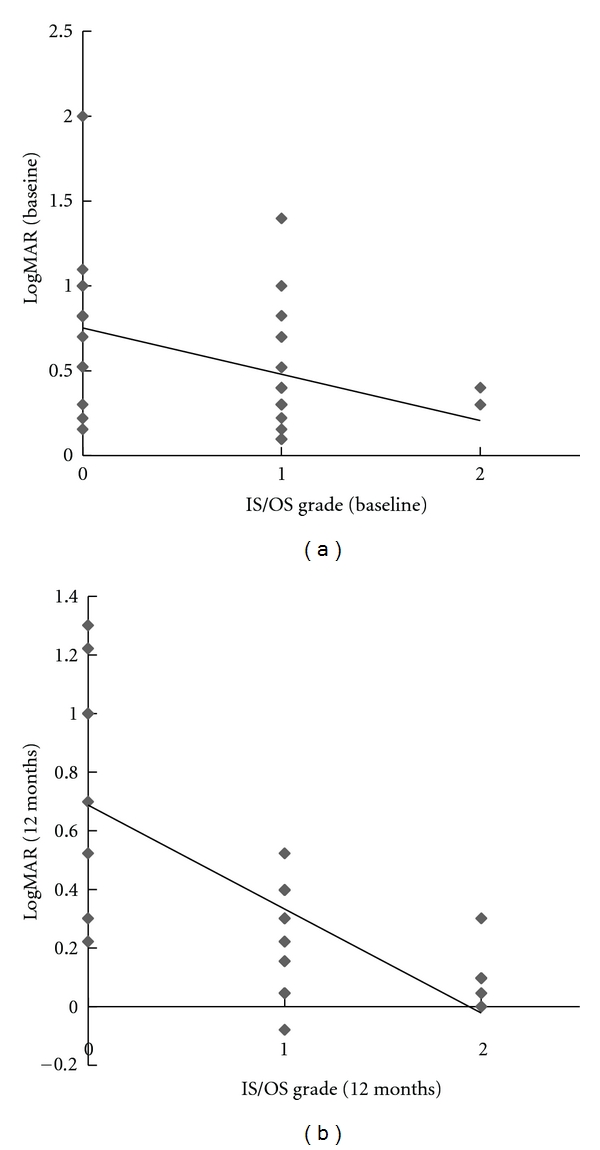
Scatter plots showing correlations between visual acuity and grades of the inner and outer segments of the photoreceptors (IS/OS). Visual acuity was expressed as the logarithm of minimal angle of resolution (logMAR). (a) Scatter plot at baseline. There was a significant negative correlation between visual acuity at baseline and IS/OS grade at baseline (Speaman correlation coefficient [*r*_*s*_] = −0.429; *P* < 0.01). (b) Scatter plot at 12 months. There was a significant negative correlation between visual acuity at 12 months and IS/OS grade at 12months (*r*_*s*_ = −0.687; *P* < 0.01).

Figure 3 showed scatter plots of BCVA at baseline and 12 months in 3 grades of IS/OS line. At baseline, IS/OS line was evaluated in OCT images of 38 eyes, which were divided into 16 eyes (42.1%) of grade 0 of IS/OS line, 20 eyes (52.6%) of grade 1 of IS/OS line, and 2 eyes (5.3%) of grade 2 of IS/OS line (Figure 3(a)). At 12 months, IS/OS line was evaluated in OCT images of 28 eyes, which were divided into 8 eyes (28.6%) of grade 0, 14 eyes (50.0%) of grade 1, and 6 eyes (21.4%) of grade 2 (Figure 3(b)). The grades of IS/OS line negatively correlated with BCVA at baseline and 12 months (*P* < 0.01), respectively. There was no significant correlation between the grades of IS/OS line at baseline and BCVA at 12 months.

### 3.5. Adverse Events and Complications

ICGA was performed in 31 of 38 eyes at 3 months to evaluate choroidal hypoperfusion according to a grading scale used in a previous study [16]. Among 31 eyes, 4 (12.9%) showed no effect on the choriocapillaris in early or late ICGA (grade 0), 13 (41.9%) showed mild (not significant) nonperfusion in early ICGA (grade I), 11 (35.5%) showed moderate nonperfusion in early ICGA (grade II), and 3 (9.7 %) showed significant nonperfusion in early ICGA (grade III).

There were no ocular complications such as endophthalmitis, sustained ocular pressure increase, or subretinal hemorrhage. No systemic side effects related to treatment were encountered in this study.

## 4. Discussion

Combined therapy of PDT and ranibizumab for CNV secondary to exudative AMD has been examined by several groups and has been shown to improve visual acuity and to reduce the number of intravitreal injections of ranibizumab compared to ranibizumab monotherapy [5–7, 9–13]. With combined therapy of PDT and ranibizumab, we showed that the mean visual acuity was significantly improved from 0.58 ± 0.40 to 0.38 ± 0.39 logMAR units at 12 months and that the BCVA of 36 of 38 eyes (94.7%) was improved or maintained from the baseline at 12 months. The mean CMT was significantly improved from 414 ± 120 *μ*m to 240 ± 91 *μ*m at 12 months. The TORPEDO trial, which examined the effects of combined PDT and ranibizumab injection on the same day, showed that the mean visual acuity improved by 7.2 letters and 84% of the patients had stable or improved vision at 2 years [12]. It also showed that the mean CMT decreased by 146 *μ*m [12]. These results were comparable to ours although differences in study design and sample size prohibited direct comparison. 

We showed that 57.9% of the patients with exudative AMD successfully achieved scarring without further treatments by a single PDT and 2 IVR during 12 months. Only 4 eyes (10.5%) in this study showed a recurrence of exudative changes. Mataix et al. reported that 39.6% of patients were successfully treated with only a single initial dose (PDT and ranibizumab) and remained stable for 12 months [10]. Thus, combined treatment of PDT and IVR might be sufficient to scar the CNV lesion in about half of exudative AMD patients. 

In the FOCUS study, the patients received standard-fluence PDT on an as-needed basis along with monthly ranibizumab injections. As a result, the mean sessions of PDT was 1.32 and 90.5% of the patients had maintained or improved visual acuity at 1 year [9]. In our study, the mean sessions of PDT was 1.1 and 94.7% of the patients maintained or improved their visual acuity at 1 year. Ranibizumab monotherapy for exudative AMD required 5.2 ± 2.8 IVR during a followup of 12 ± 4.3 months when ranibizumab monotherapy was performed on an as-needed basis [13]. Mataix et al. showed results of combined therapy of PDT and ranibizumab: in that study, 92.3% patients showed improved vision or at least avoided moderate loss of vision over 1 year with 1.22 PDT sessions and 2.37 IVR [10]. Their results were similar to our results even though they performed IVR within 48 to 60 hours after PDT. By combined therapy with PDT, we could limit the number of IVR to 2.9 ± 1.3 injections per patient during 12 months. 

Our protocol of the primary therapy consisted of 2 IVR and a single PDT. We performed the first IVR one week before PDT. The reasons why IVR was preceded to PDT were as follows. (1) In case that a bacterial endophthalmitis is occurred after IVR within a week, the patient must immediately need intensive antibacterial treatments. (2) Preceding IVR is reasonable to quench VEGF in the retina and the choroid after application of PDT, which was shown to induce VEGF production [17]. Sato et al. reported beneficial effects on PCV with a combined therapy of intravitreal injection of 1.25 mg bevacizumab 1 week before PDT [14]. Kaiser suggested that the intravitreal injection of bevacizumab before PDT might have beneficial effects on the retreatment rate in the combined therapy for AMD [18]. They showed lower retreatment rates after combined therapy in patients who received intravitreal bevavizumab before PDT compared to those who received PDT first or intravitreal bevacizumab and PDT on the same day [18]. 

Moutray et al. reported the relationships between GLD and visual functios in patients with exudative AMD. They showed GLD and visual acuity significantly correlated with each other [19]. Arias et al. examined the lesion size on photodynamic therapy with verteporfin of predominantly classic lesions [20]. They reported that the group of smaller lesions had more improvements with one or more lines of visual acuity compared with the larger lesion groups. In this study, we showed a similar tendency in the improvement of mean BCVA of all eyes, typical AMD, and PCV. The smallest GLD group showed better mean BCVA during 12 months although there was no significant difference among the 3 groups of GLD. The mean CMT showed a significant decrease in all 3 groups of GLD during 12 months, but there was no statistical difference among the 3 groups of GLD. The mean CMT did not show a clear tendency according to the GLD size. 

Several studies reported that the presence of a normal IS/OS junction indicated normal functions of photoreceptors and the grades of IS/OS line correlated with visual acuity [21, 22]. We showed that grades of IS/OS line significantly correlated with the BCVA at baseline and 12 months, respectively. Better IS/OS line indicated better visual acuity, however, grades of IS/OS line at baseline did not correlate with the BCVA at 12 months. The grade of IS/OS was not shown a predictive factor of BCVA in this study.

We also examined choroidal hypoperfusion with ICGA at 3 months after combined therapy of PDT and IVR. Fourteen of 31 eyes (45.2%) showed moderate (grade II) or significant (grade III) nonperfusion in early ICGA. Iriyama et al. showed that standard-fluence PDT (50 J/cm^2^) caused a significant loss of choriocapillary perfusion in 40.9% of the patients [23]. Michels et al. showed that 60% of patients treated with standard-fluence PDT (50 J/cm^2^) had moderate or significant choroidal nonperfusion changes in early ICGA at 3 month [16]. In this study, we did not examine changes in choroidal hypoperfusion between 3 months and 12 months. Further studies are needed to examine effects of lower fluences of PDT on choroidal hypoperfusion and effects of ranibizumab on lasting periods of choroidal hypoperfusion. 

Combined therapy of IVR and PDT seemed effective on both typical AMD and PCV for a short term, however, randomized clinical trials are needed to show which combination of IVR with PDT is the most effective for typical AMD and PCV on improvements of visual functions over the long term.

## 5. Conclusions

PDT combined with IVR showed a siginificant improvement in visual acuity and in CMT for exudative AMD of Japanese patients with low recurrence rates for 12 months. Grades of IS/OS line was significantly correlated with visual acuity. Further studies with longer followup periods are necessary to assess treatment safety and efficacy.

## Figures and Tables

**Table 1:** Treatments during 12 months.

Mean PDT sessions	1.1 ± 0.3 (1-2)
Mean IVR	2.9 ± 1.3 (2–6)
Total number of combined treatments	
1 PDT + 2 IVR	22 (57.9%)
1 PDT + 3–5 IVR	11 (28.9%)
2 PDT + 4–6 IVR	5 (13.2%)

IVR: intravitreal injection of ranibizumab, PDT: photodynamic therapy.

**Table 2:** Recurrence rates of exudative changes after successful initial treatment.

Initial treatment	Eyes (%)	Recurrence eyes (%)
1 PDT + 2 ranibizumab	23 (60.5%)	1 (4.3%)
1 PDT + 3 ranibizumab	9 (23.7%)	2 (22.2%)
1 PDT + 4 ranibizumab	2 (5.3%)	1 (50.0%)
1 PDT + 5 ranibizumab	2 (5.3%)	0 (0.0%)
2 PDT + 6 ranibizumab	2 (5.3%)	0 (0.0%)

Total	38 eyes	4 eyes (10.5%)

PDT: photodynamic therapy.

**Table 3:** Changes in visual acuity after PDT combined with IVR during 12 months.

Changes in logMAR from the baseline	1 month	3 months	6 months	12 months
≧0.3 logMAR units improvement	7 eyes (18.4%)	16 eyes (42.1%)	14 eyes (36.8%)	15 eyes (39.5%)
Changes under 0.3 logMAR units	30 eyes (78.9%)	21 eyes (55.3%)	23 eyes (60.5%)	21 eyes (55.3%)
≧0.3 logMAR units deterioration	1 eye (2.6%)	1 eye (2.6%)	1 eye (2.6%)	2 eyes (5.3%)

IVR: intravitreal injection of ranibizumab, PDT: photodynamic therapy.
